# Innovative Strategies to Fuel Organic Food Business Growth: A Qualitative Research

**DOI:** 10.3390/ijerph19052941

**Published:** 2022-03-03

**Authors:** Sonia Chien-I Chen, Chenglian Liu, Zhenyuan Wang, Farid Arya

**Affiliations:** 1 School of Economics, Qingdao University, Qingdao 266071, China; drsoniachen@mail.com; 2 Department of Science and Engineering, Shiyuan College of Nanning Normal University, Nanning 530011, China; chenglian.liu@gmail.com; 3 Faculty of Economics and Management, East China Normal University, Shanghai 200050, China; 4 School of Engineering, Buckinghamshire New University, High Wycombe HP11 2JZ, UK; farid.arya@bucks.ac.uk

**Keywords:** organic food, sustainable consumption, purchase intention and availability, market-led innovation

## Abstract

This study aimed to identify the factors affecting consumer behavior and customer loyalty toward organic food. Whether consumers seek organic food for a healthy body or more as food for thought continues to be debated. However, since consumers’ purchase habits are based on their honest life experiences, which shape the building of a brand, this study reviewed the extant literature to understand the factors influencing the purchasing behavior for organic food. The follow-up problems highlighted in the research are related to organic business marketing strategy. Based on our methodology, we conducted semi-structured interviews to gain themes for qualitative research. The study found that availability, variety, and taste were the top three factors affecting consumers’ purchase decisions; surprisingly, neither price nor health-consciousness was the first concern. Using market-led innovation as an innovative lens to understand customer loyalty, this research highlights sustainable and advantageous business practices in the organic food market to enrich the literature on organic food purchasing behavior from multiple stakeholders.

## 1. Introduction

With health and safety consciousness accompanying improvements in socioeconomic status [1], tasty, healthy, and trendy eating has gradually replaced the notion of “eating to live” [2,3]. Consumers are increasingly demanding better quality and more environmentally friendly products [4,5]. Consequently, organic products have become a popular trend among the middle to higher socio-economic classes seeking a sustainable and healthy lifestyle. However, whether organic food is food for the body or food for thought remains debatable.

Organic farming is a sustainable agricultural method that helps preserve soil fertility to produce chemical-free products. While there are opposing views on organic farming due to its high bacteria levels, those in favor of organic farming believe that it is a beneficial and safer alternative for the environment and animals. However, it is not an option for everyone, and yields are often insufficient to feed the majority of the population. Although there is an increase in sales of organic food each year, it is still limited to certain market segments and does not contribute a large proportion of steady purchases [6]. The sustainability concept of organic food is interpreted as an idealism that drives it beyond food, to fashion [7,8].

As the literature on organic food is focused on food, agriculture, and environmental sciences, studies on consumers’ purchase intentions and behaviors are growing as people shift toward adopting a healthier lifestyle [9,10,11,12,13,14]. The theory of planned behavior (TPB) is a popular framework that helps understand and explain purchase intentions in light of social norms [15,16]. Subjective norms are found to be associated with attitudes toward organic food purchase intentions. Nonetheless, the inconsistency of actual purchases and the relationship between norms and purchase intention drives research direction to factors such as personal choice, barriers, and consumers’ abilities. In this context, innovative marketing strategies may be relevant in meeting diverse purchase intentions.

There has been an increase in research on organic food consumption, with a few of them focusing on the Irish market, which has seen significant growth recently from being a small-scale producer to an important market segment in the mainstream agri-food system. This change has attracted the attention of both agri-business and brand retail investors. Although researchers have studied the habitual and occasional pro-environment behavior of organic food consumers in Ireland, a qualitative method explaining purchase behavior and customer loyalty is still lacking [17]. The organic products business in Northern Ireland (NI) faces an expanding market, but many small businesses are still struggling to identify an appropriate marketing strategy. To obtain better insights, this study employs a qualitative approach to the investigation of the Ireland/Northern Ireland organic market. Based on the literature review, which indicates a lack of innovative theories, this study sheds light on applying a market-led innovation concept to promote customer loyalty through strategy, system, and people [18]. It aims to contribute to the understanding of customers’ purchase intentions in the organic market by providing insightful information on customer loyalty, which may assist agribusiness developers and practitioners in improving and optimizing marketing strategies. 

## 2. Material and Methods

This study explored customers’ purchase intentions through semi-structured interviews (*n* = 20). Due to the infinite and flexible characteristics of consumer behavior, qualitative research was chosen to generate a better understanding of the growth and success of organic businesses [19,20]. In view of the controversy on the health properties of organic products, in-depth interviewing was seen as a crucial method of qualitative research. This study focused on identifying the factors that influence consumers’ purchasing behavior and interpreting the values behind them. It also explores the current market situation by asking “how” and “why” questions. 

### 2.1. Research Design

**Figure 1:**
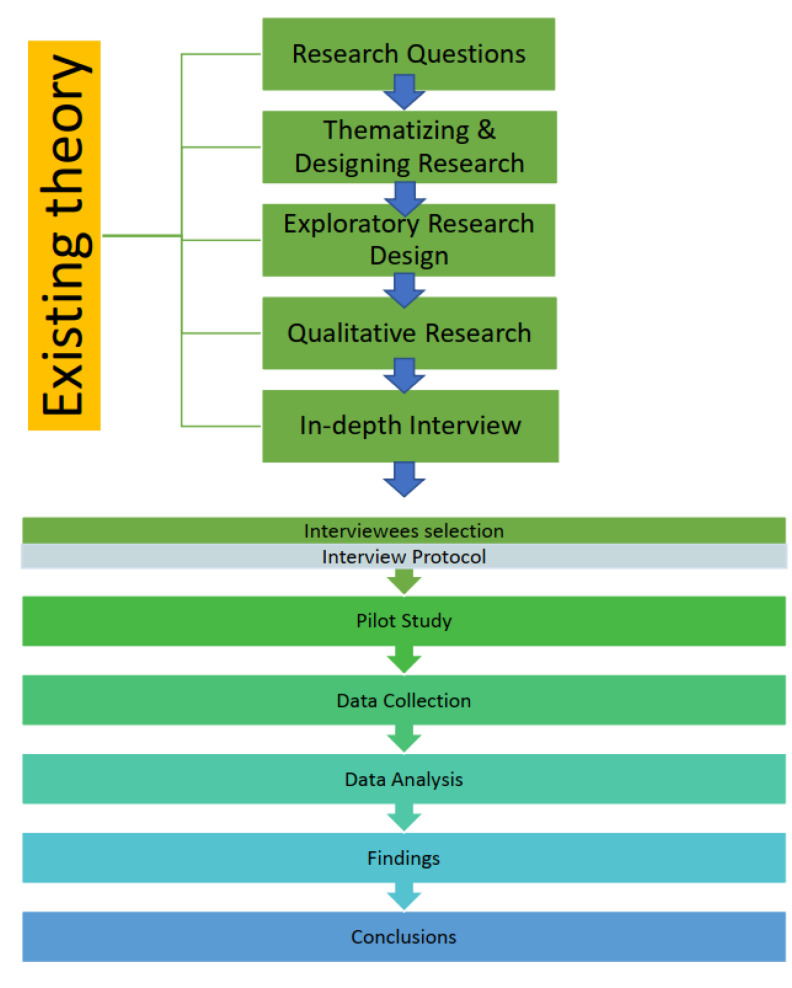
Research design conceptual framework.

The research framework in Figure 1 guided data collection, measurement, and analysis, and builds on current knowledge and the existing theory for organic food. This study consists of several phases. First, potential participants in the Northern Ireland organic food ecosystem, identified in the food business circles and approached through friends and contacts online, were selected as interviewees through a snowball sampling technique, after which the interview protocol was developed. Second, a pilot study was conducted to evaluate the feasibility, duration, and cost of the study, based on which improvements were made to the study methodology and in-depth interviews were conducted. Third, based on the interviews, data and information were collected on various stakeholders, such as suppliers, producers, consumers, and experts. Fourth, data analysis was conducted, by cleaning, transforming, and modeling the data to find answers to the research questions. Finally, qualitative research was used to interpret market understanding, multiple realities, different perspectives, and specific circumstances arising from opinion-sharing, interview discussions, data analysis, findings, and final discussions. Based on this, conclusions and recommendations were drawn.

### 2.2. Data Collection

Identifying the segments from which the interview sample can be drawn ensures consistency with research aims and questions. The relevant research indicates that the main motivations for purchasing organic products are health, taste, and concern for the environment, and that older, more affluent consumers are the main purchasers [4,5,6,7,8,9,10,11,12]. The above motivations were used as criteria to determine the status of the interviewees. The interview questions were designed with reference to the literature through key themes affecting organic business. This adjustment is essential to obtain a deeper understanding of interviewees’ thoughts about organic products. A pilot study and consultation with experts were conducted to guide the answers to the interview questions. Then, the selected interviewees were contacted through friend referrals and social networks to arrange e-mail or phone interviews. 

In this phase, we aimed to uncover the reasons behind customers’ intention (or lack thereof) to purchase organic food and how food suppliers viewed future trends in the organic business. Manufacturers were interviewed to determine how they currently marketed their businesses. Suppliers, producers, consumers and experts related to organic products were invited to join in an informal conversation about eating habits and purchasing behavior. The owner of Burren Organic Farm was chosen to review the significance of the marketing strategy for organic food. Sales managers from local suppliers in Northern Ireland were invited to contribute their views on the local organic business. The owner of a new local shop participated to see how a small business could use innovation marketing to survive in a changing economic environment. By collecting different perspectives, this research aimed to inspire more creative ideas for innovative advantages in the novel agri-food business. A total of 20 interviews were conducted with producers (1), suppliers (5), experts (2), and consumers (12) (Table 1).

For the face-to-face interviews with 20 participants, which lasted for approximately two hours, the principles of informed consent were followed. Semi-structured interviews were conducted to allow for flexibility for the interviewees to openly express their opinions. The interviews were audio-taped and transcribed by an independent typist and subsequently validated by the researcher. Meaningful quotations were used to identify significant themes. All considerations for maintaining anonymity were applied to ensure confidentiality. 

**Figure 2:**
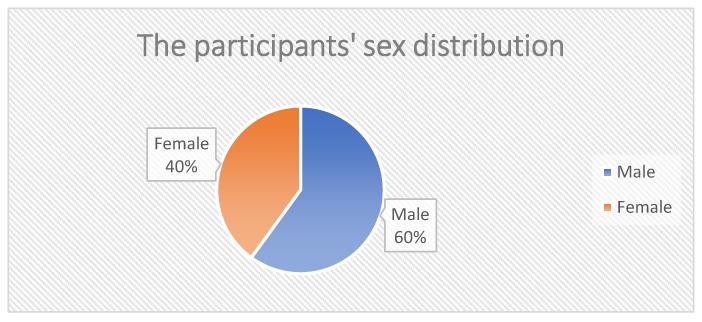
The participants’ sex distribution.

**Figure 3:**
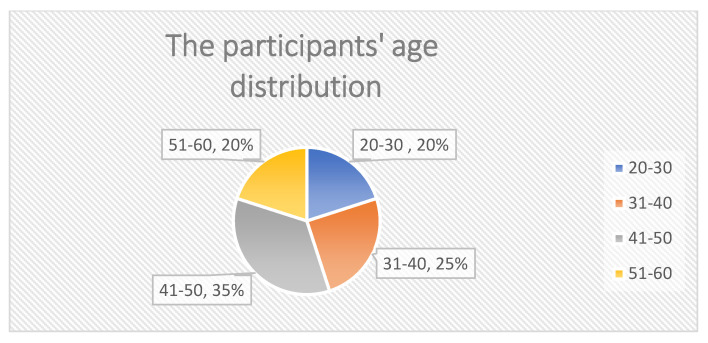
The participants’ age distribution.

**Figure 4:**
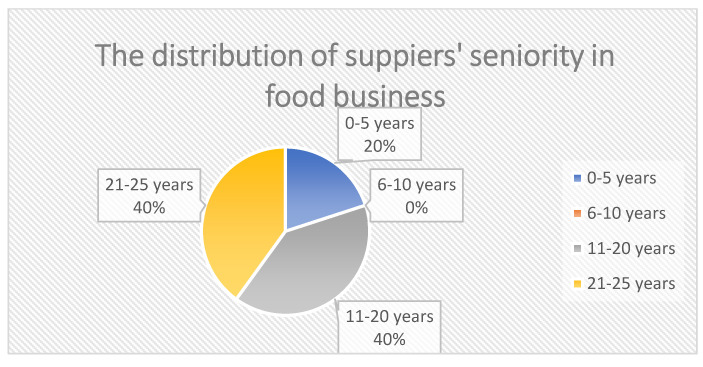
The distribution of supplies’ seniority in the food business.

Figure 2 illustrates the participants’ sex distribution. There is only a small difference in sex distribution of the participants of this study, as shown in Figure 2. The percentage of male interviewees was slightly higher than the percentage of female interviewees (which accounts for 60% and 40%, respectively). As seen in Figure 3, participant age distribution was centric, between 31–50 years old, and the majority of participants were between 31 and 50 years old, accounting for 60% of the total. The percentage of participants over 51 years old was the same as that of participants of age under 30, each of these accounting for 20% of the total. Figure 4 illustrates that most suppliers selected had over 10 years of experience in the food business, with only 20% of them having under 10 years of experience. 

### 2.3. Data Analysis

**Figure 5:**
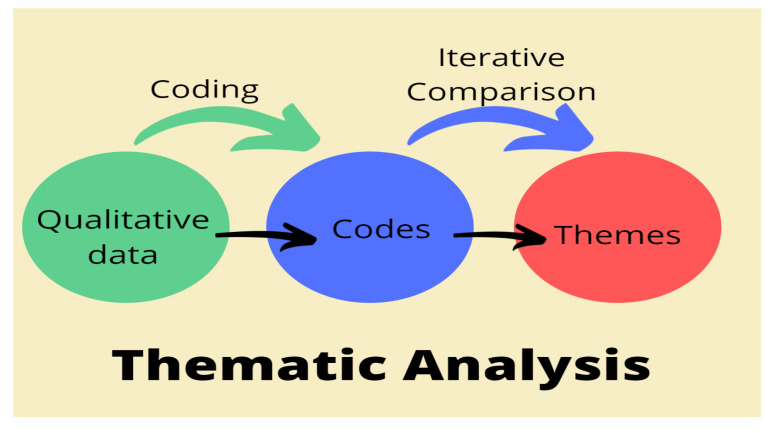
Thematic data analysis diagram.

Interview data were organized into key themes through coding processes and iterative comparison as shown in Figure 5. Follow-up interviews were conducted as and when necessary. The data were cleaned and transformed to extract valuable information for business intelligence acquisition. Pragmatic information was extracted for better decision making. A thematic approach to qualitative analysis was performed through deep familiarization with the data collected, and codes were extracted through coding, leading to the development of major themes focused on interviewees’ opinions, as shown in Figure 5. These themes were further analyzed to uncover sub-themes and were structured for a comprehensive account. An iterative comparison was continuously performed until the themes and categories accurately reflected the information. In data cleaning, different terms with the same meaning, based on what the interviewees intended to say, were merged. Then, the data were summarized, clustered, and categorized based on the influencing factors specified in the research questions and then analyzed to generate insights.

### 2.4. Validity

Two strategies were employed to establish validity: data blinding and data triangulation. The research comprised knowledge of organic food purchasing assessment among the present population of Northern Ireland. The sample was separated into several groups to reduce biases. The sample engaged potential consumers who purchased or were likely to purchase organic food, along with suppliers, producers, and related experts. The inclusion of multiple perspectives reduced bias toward the outcome, creating a base for valid results. The other technique adopted was to confine the amount of information shared with the respondents to ensure that the research was not biased by their predetermined notions. These steps aided in ascertaining the validity of the results gained, proving the accuracy of the qualitative research. Further, the validity of the questionnaire was established with the help of a panel of experts who reviewed the questionnaire. Consequently, inappropriate statements were removed. 

### 2.5. Reliability

Two processes were conducted to boost reliability. First, different investigators ensured consistent results were obtained, indicating data reliability. Second, relevant literature was analyzed to support the claims of the data collection and analysis process. Careful and systematic data encoding is crucial in ensuring the efficiency of data checks. Moreover, the reliability measures relating to data triangulation provided an extensive understanding of the research aims and objectives.

## 3. Results

### 3.1. Descriptive Statistics of the Participants

**Figure 6:**
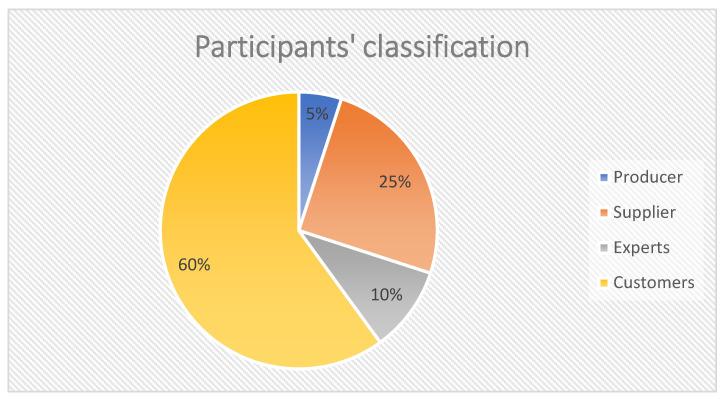
Distribution of participants by category.

There was a total of 20 participants from 17 meetings, representing all types of stakeholders in the organic food ecosystem (Figure 6). Most participants were customers (*n* = 12; 60%), including organic food customers and its potential customers, and suppliers *(n* = 5, 25%), accounting for more than three-quarters of the sample. In addition, there were two experts (10%) and one organic producer (5%). 

#### 3.1.1. Customers’ Profile

**Figure 7:**
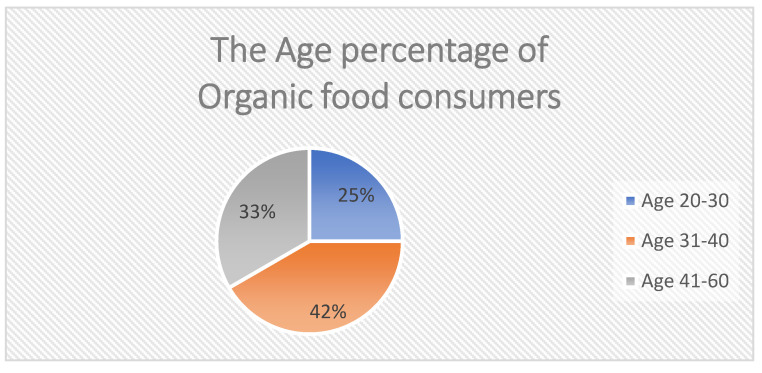
Distribution of organic food consumers by age.

The customer profile in terms of age, sex, marital status, employment, and health, and organic food consumption is described in Table 2 and Figure 7. The consumers were mostly Irish residents, with university level and above-average education and comprised eight nationalities, aged 24–58. Most of the customers were men (60%), but there was no significant difference in the number of male and female participants. The majority belonged to the 31–40 age group (40%). The second highest age group (41–60) accounted for one-third of the sample size. 

#### 3.1.2. Analysis of Consumer Responses

The interview questions were designed to provide insight into how marketing strategies could be formulated through an understanding of consumer behavior. Thus, the analysis focused on consumers’ opinions obtained from responses to the following:

Q1: What are the significant factors in food purchasing?

Q2: What are the motivations for purchasing organic food? Are price and availability barriers to purchasing organic food?

Q3: What are the barriers to purchasing organic food? Does knowledge of organic food affect the motivation for purchasing organic food?

Q4: Do people care about the environment?

Q5: Do people have a sufficient understanding of organic food?

The interview data are summarized in Table 3. Influencing factors such as availability, price, preference, eating habits, health concepts, and taste were extracted from the interview transcripts. Motivations for buying organic food were health concepts, eco-friendliness, taste, and cost-effectiveness. In contrast, barriers to purchases were availability, price, culture, insufficient knowledge, and food security. Most respondents connected ecological concerns with organic food. It seems that most interviewees required adequate knowledge about organic foods. 

#### 3.1.3. Analysis of Producers’ and Suppliers’ Responses

Regarding the producer and suppliers’ perspectives on organic food selling, the following questions were asked in the interviews:

Q1. What are the motivations for marketing food?

Q2. What are the barriers to the marketing of organic foods? 

Q3. Do food marketers care about the environment?

Q4. Do food marketers have sufficient knowledge of organic food?

Table 4 summarizes suppliers’ opinions according to their motivation, barriers, ecological concerns, and knowledge of organic food. Health concepts and profits motivate suppliers to sell organic food. Barriers to selling organic food were premium prices, consumers’ eating habits, and comprehensive knowledge of organic food. Most suppliers support the ecological concept of organic food but admit that they do not have sufficient knowledge about it. 

### 3.2. Influencing Factors and Motivations 

This section summarizes the findings of the interviews. Several key themes and some direct quotes from interviewees support these arguments. 

#### 3.2.1. Why Do Customers Buy the Goods or Services You Provide?

The respondents were interviewed based on their observations on general consumer purchasing behavior. The major themes are as follows:

- Convenience/Availability/Endurance

Distribution plays an important role in the delivery channel between manufacturers and consumers.

S-1 stated: “Another key thing is your distribution; you have got to have products on every shelf… for example, Kellogg’s is famous… Heinz has three or four products on the shelves. Why do they do so well? Because people see them everywhere they go, and so form an emotional attachment. This is the result of the manufacturers’ marketing strategy, which creates a huge demand for their products in every outlet.”

C-2, a householder, noted that convenience was the most important factor for her purchase. C-1, a consumer who prefers organic products, believed “The availability comes first… then the affordability; if readily available, more people will consume it.” Andrew, the owner of a local shop, noted that “Location is very important.” Their testimonies suggest that greater availability should increase sales. “So, distribution… needs to be built, because you want to list in high volumes to get the high turnover. Well, one thing that drives it is… can the buyers buy it in a local shop?” Similar points such as shelf life and product endurance were also emphasized. Both S-1 and C-2 agreed that shelf life was important.

1. Quality/Taste/Variety/Attractiveness

Product quality is an important factor in purchasing; for many, it may be among the top three factors in purchasing. S-2 believed that quality food could be an opportunity for small businesses to innovate and produce quality products. Yet, he claimed that “organic meat doesn’t taste any better than his (quality) meat.”

- Brand/Source

Branding is crucial for businesses as it creates value and emotional attachment with customers. Consumers place their faith in the most trusted brands. S-1 considers brand trust as a key reason people buy a product and the reason why many companies concentrate on branding. Many studies have focused on brand marketing [21,22]. C-9 and C-1 said they purchased products only from what they considered a reliable source.

- Price

Price was mentioned by many consumers, including S-1, who argued: “At the moment in the current market, price promotions are ruling the roost. They are the keys to your business… and if you are not in there, your business will struggle. You have to price accordingly to allow you to market your product on the shelf… through price reduction, driven by the current economic climate.” However, it is not always the priority; as C-5, C-12, and C-1 state, other factors such as quality or health are superior to price. Usually, middle-aged consumers with above-average education do not care much about the price. S-1 claims that “Price is not the priority, and price here does not mean cheaper, it means the right price, it may be in the top ten or (even) the top four factors at the most.” C-12 also mentioned in the interview that “Organic food is my favorite… Price is not the first concern for purchasing …I always buy them without noticing the price.” However, S-3 argued that “Organic food markets are in decline, as the recession has led consumers to be more careful about their expenditure on food and they may not be prepared to pay the premium price associated with organic food.” C-7, a semi-retired teacher, states, “We don’t buy organic as it is too expensive!”

- Health

Health issues are a popular trend in businesses today. S-1 argues that “Food can feed people; it can also kill people; many diseases like diabetes and obesity are the result of a poor (quality) diet.” C-8 also mentioned that his purchase decision is influenced by his health concerns: “I am mainly concerned about salt and especially sugar content because diabetes is a terrible disease!” He is an example of a young and highly educated representative. C-3, a German consumer who just graduated from university, thinks that “people should take care of their bodies.” C-8 believes “You are what you eat!” The problem of obesity and health concerns prompted him to start eating healthier. “The side effect of chemicals in food or medicine needs to be considered.” S-1 stated that “freshness and health will be the future trend in the food industry… For competitiveness in the food industry, we need to be innovative… However, there is always a balance between health and affordability.”

- Environment Friendliness/Social Responsibility

Most organic consumers and vegetarians have a strong scientific awareness. Both individuals and corporations pay much more attention to social responsibility these days. C-4 claims: “I will try my best to reduce energy or package waste.” C-5, C-8, and C-9 said they would recycle as much as possible and try not to waste energy. Many companies are willing to exploit social responsibility in their marketing strategies. S-3 said: “All companies are concerned about corporate responsibility and are more aware of things like carbon footprint. We as a business are also mindful of the social impact of specific marketing campaigns, and take this into account when considering any new product development or specific campaigns.”

#### 3.2.2. What Do Customers Really Value?

**Figure 8:**
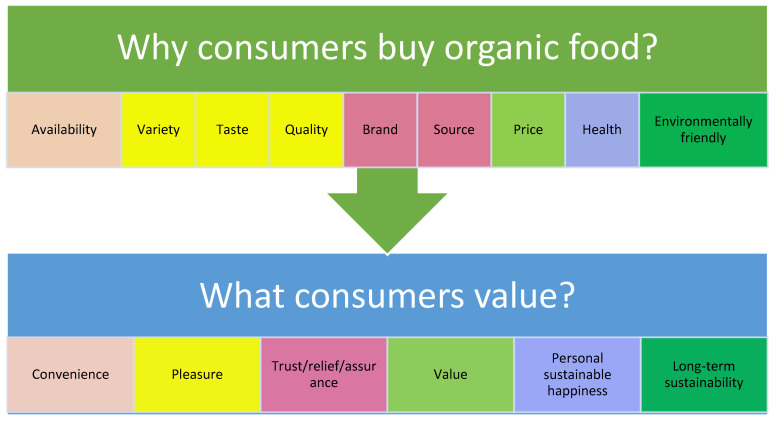
Features most valued by consumers.

The results are analyzed according to the question of what customer value, which is summarized to present how results are interpreted as significant themes (as shown in Figure 8).

- Convenience/Efficiency/Consideration

Today, everything is expected to be “fast.” Therefore, although people mention availability, shelf-life, or product endurance, they expect to obtain something efficiently or conveniently.

- Pleasure

Consumers look for the quality of life rather than only survival. Hence, they expect food to be aesthetically appealing, of better quality and taste, and having variety. A significant attribute of food is its ability to impart mental satisfaction, what we call pleasure. C-2 emphasized it when she said she would not buy food solely because it was healthy; it must be aesthetically pleasing and appealing enough to persuade consumers to make a purchase.

- Trust/Assurance/Reliability

The results reveal a consideration for brands or sources during purchase, as interviewees believe they give assurance of quality products that they can trust. Hence, in buying branded goods, people mostly pay for assurance and reliability.

- Value/Worth

This section seeks to discover what is behind price. Arguing that “organic food is better than artificial foods because of the latter’s adverse side effects,” C-1 talked about the long-term consequences of his unhealthy dietary pattern before he switched to organic food; his account highlighted the long-term health benefits of consuming organic foods. Furthermore, S-1 noted that consumers are willing to pay the right price and do not necessarily choose the lowest price. For example, C-12 claimed that she would not buy “Tesco’s Value Meat.” She goes to local shops to buy more expensive quality meat. These statements prove that people pursue products of value, and price is not the sole factor in the decision-making process. When they consider that the price too high, they refer to the value of products that must be appreciated and recognized. Therefore, marketers should set the right price to make customers feel that the product is worth their money.

- Sustainable Personal Happiness

Customers value healthier food to achieve happiness. E-1, a human nutrition student from a food standard agency, argued: “Health should be the priority… If you ask someone who suffers from a disease, they would tell you that there is no happiness without health.” However, C-1 would not value health unless he suffered an adverse health condition.

- Long-term Sustainability

Ecological issues or social responsibility can be interpreted as long-term sustainability, as it is a value that is appreciated over a period. In other words, if businesses want to be sustainable in the long run, they cannot disregard the importance of environmental sustainability.

### 3.3. Barriers to the Purchase Decision

The respondents were identified as potential customers of organic foods and were assumed to be interested in organic food. However, not all of them buy organic products, and the reasons can be attributed to several factors according to the interviewees’ responses. In this section, the barriers to organic purchasing are presented: price premium, availability, and attractiveness.

#### 3.3.1. Price Premium

Respondents C-7 and C-6 are potential organic food customers, as C-7 has obesity and heart problems, and C-6 has a nursing background. However, they do not frequently shop for organic products. C-7 noted: “The price is too expensive… Organic food belongs to the high-medium class.” C-6 hoped: “Maybe we will buy it after the children are married and have their own family.” C-3 and C-4 have healthy eating habits, yet they both claim that they buy organic food occasionally when they can afford it. C-8 mentioned: “I have experience with organic food, but I don’t really buy it often because it is more expensive.” S-3 argued: “Consumers are not convinced of the benefits of organic food and are not prepared to pay the premium for it. The recession has seen consumers switching buying habits away from premium, high-value foods where organic foods sit. Consumers are seeking value for money now and buy what they need.” C-5, an organic food consumer, stated: “The price of organic food can also cause me to think twice and make me ask myself if it is really worth it.”

#### 3.3.2. Availability

As S-1 mentioned, if one repeatedly sees a certain product in shops or through various marketing channels, one automatically forms an attachment to the product. C-1 added that although he was really interested in organic products, he was not sure where to purchase them, apart from his usual stores such as Tesco, Holland, and Bernard.

#### 3.3.3. Attraction

The benefits that organic foods offer to humans and the environment are not enough to render them appealing to the customers. C-2 stated that “I will not buy it simply because it is healthier.” If organic products are placed along with other standardized products, they must be advertised or highlighted specially to make them more appealing and allow customers to distinguish them from other products. C-3 maintained, “I will buy it only when they have a promotion.” Marketers need to advertise organic products to enhance their attractiveness and convert customer interest into purchases. 

### 3.4. Products Knowledge

#### 3.4.1. Consumers’ Perspectives

Among the respondents, most of the organic consumers are teachers or lecturers in schools. When asked about the understanding of organic food, common replies are “Organic food is natural, healthier and good for the environment.” This is correct but not sufficient. C-5, an organic food consumer, said, “I …purchased organic food in the past, but there are so many arguments about its value as opposed to non-organic foods that I am now undecided. I know what I have heard on TV and radio, which I suppose is not much. I have not attended any lectures or read articles about these types of food.” C-8 mentioned, “I think not eating a lot of meat is better than buying organic food. Of course, both would be best. I know that organic food is environmentally friendly. I also believe that animals are at least supposed to have a better life. The thing that concerns me is the safety of organic food because some things like pesticides are not used. I think enterohemorrhagic *Escherichia coli* (EHEC bacteria) is a risk in consuming organic food.”

#### 3.4.2. Suppliers’ Perspectives

S-1 was of the opinion that organic products are considered product innovations that respond to the current trends in the demand for healthier and higher-quality products. The science behind organic food, or the definition of organic food, is of less importance to S-1 than sales and profit. Similarly for S-3, what matters are profits and business benefits, and therefore, he tends to promote conventional products to please more customers. S-2 claimed he sought to offer quality products for business benefits, irrespective of whether these were organic products. When it comes to selling, he would pay attention to healthier and more natural trends.

#### 3.4.3. Producers’ and Suppliers’ Perspectives

**Figure 9:**
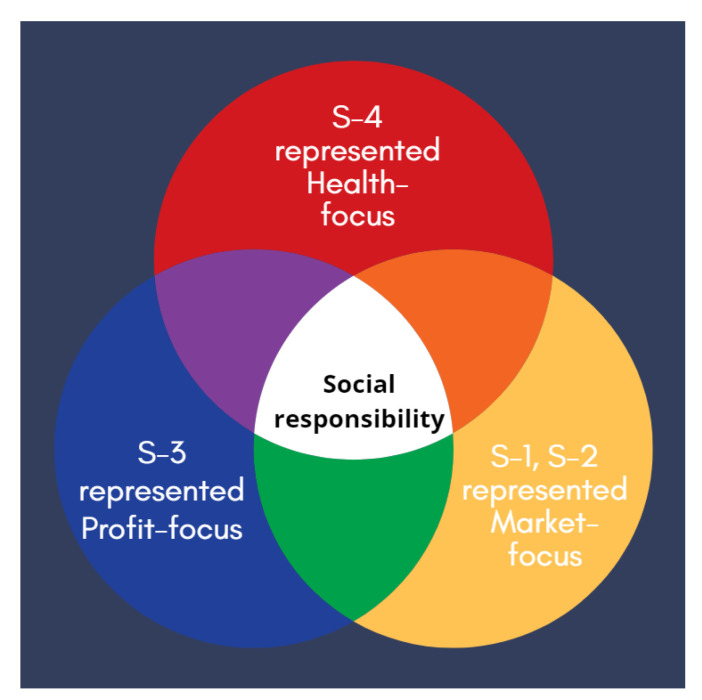
Comparison of focus between suppliers and producers.

Undoubtedly, producers have the most accurate and competent understanding of organic products. They not only consider the products themselves, but also all manufacturing processes. Suppliers can be divided into three types based on their attitude toward the market: health-focused, profit-focused, and market-focused, as shown in Figure 9. S-4, an organic supplier and producer, plays a health-focused role, as his business concern is based on health, while S-1 and S-2 will observe market trends to adjust their business direction and lie between health-focused and profit-focused suppliers. We call them market-focused suppliers, as they pay keen attention to quality and health products. S-3, a non-organic supplier, pays attention to cost and profit concerns, making him a profit-focused supplier. While profit-focused suppliers view business differently from market-focused and health-focused suppliers, they pay attention to social responsibilities as well.

- Education of Consumers

Only the producer is aware of the differences between organic and non-organic products. It seems that customers do not have sufficient understanding of organic products. Therefore, there is a need to educate people and provide them with accurate information. As C-2 stated, “If someone told me the specific benefits of organic food, I will buy it.” Both C-1 and S-2 agree that education is important.

- Communication with Customers

Consumers do not possess the same level of knowledge as producers, as their information is mostly sourced from mass media and newspapers. Thus, if the media provides a negative report on organic products, it will affect their confidence in consuming them. Therefore, producers must engage in proactive communication with customers. Otherwise, consumers will have to rely on information from websites or media and can be often misled.

## 4. Discussion

This section presents and discusses the findings in relation to those of previous studies, demonstrating how they confirm or complement published literature. It also discusses the implications of the findings, presents new findings with respect to marketing strategies for organic products, and indicates future research directions. 

### 4.1. Main Findings

The study explored the influencing factors and barriers to organic food purchasing, as well as the product knowledge of various stakeholders in the ecosystem shown as Table 5. It found that factors such as availability, variety, taste, quality, brand, source, price, health, and environmental friendliness affect consumers’ purchase intentions and behaviors. Consumers value convenience, pleasure, assurance, value, happiness, and sustainability (see Figure 8). Certain barriers prevent people from purchasing organic food, such as price premium, availability, and lack of appeal. Although knowledge of organic food is diverse among different stakeholders, education is helpful in promoting comprehensive understanding. Family style and globalization are interesting factors that were not mentioned in the interviews, but social responsibility is a common ground for suppliers and producers from different backgrounds. The principal findings converge to three research themes for further discussion:

1. How can value be delivered?
2. How much do customers understand the products?
3. Who are the best customers for organic products?

### 4.2. A Comparative Discussion of the Findings and the Literature

**Figure 10:**
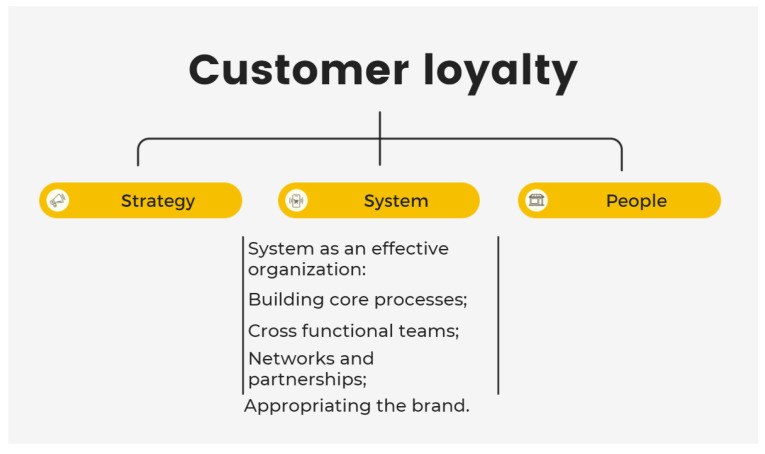
Market-led innovation: systems [18].

The model of “market-led innovation” shown in Figure 10 is used to discuss the findings, as it fulfills the aims and objectives of this study. This model suggests that strategy, system, and people are the three pillars required to maintain customer loyalty. These three elements were extracted to further discuss the three research themes mentioned above. The results of this study identify the pursuit of the current trend of better quality and health concepts, suggesting the current understanding of how innovative marketing strategies can help achieve sustainable business success. Although the price premium is considered as a critical barrier to organic food purchasing, given that customers are especially prudent in their spending when the economy is depressed, what they care for is the “value” rather than the “cost” of the organic product. If the value of the price is recognized, customers will purchase the product. Therefore, the delivery of this value will be discussed in the next subsection.

#### 4.2.1. Value Delivery

- Availability

The element of “system” from market-led innovation (shown in Figure 10) is extracted to discuss how value is delivered with the themes of availability, branding, and communication from the findings (shown in Figure 8). As mentioned before, availability is significant in promoting organic business, and people today expect more efficient access. The literature suggests that greener products should be made available in mainstream outlets [21,23]. Regardless of the excellence of a product, it is necessary to ensure its availability to consumers [23]. Unless the systems can deliver high-quality products, marketing is nothing more than an aspiration. Therefore, the role of product availability in attracting consumer demand is critical [24,25,26]. S-5 admits that their store would have better sales performance if they had a better location and logistics system. The literature also supports that improvement of nutritional quality and availability of healthy foods can promote their purchase [26]. Availability is a critical factor that impacts customers’ total purchase costs and consequently affects their consumption behavior [27,28]. In addition, S-1 agrees that the exposition of products will increase their attachment to customers, which is why many branding companies include it in their strategic planning. Thus, the ability of an organization to assemble internal and external networks to work together determines the effectiveness of its supply chain or value delivery system.

- Branding

The system theme refers to an effective organization that addresses value through core networks as well as through appropriate branding. Brand consciousness is one of the vital styles affecting consumer decision-making of organic products [29]. According to the interviews, S-1 believes in the power of branding, and C-9 also purchases branded products. If a company expects to build a strong position, brand recognition by consumers is crucial. It allows them to have power in the network and is usually suitable for most of the value created. Brand reputation influences the relationship between consumer values and attitudes. The higher the brand reputation, the stronger the motivation of consumers to buy organic food. Branding improves the value delivery of a product [29,30]. Further, both the additional constructs (environmental consciousness and health consciousness) were also found to be significant [31], enhancing the image of organic food in potentially influencing consumers’ subjective wellbeing [32].

- Communication

Communication with customers serves as an essential strategy to deliver value in marketing through advertisements or promotions as well as through the translation of images and concepts to consumers. Research suggests that consumers occasionally encounter morality calls to be green by buying environmentally friendly products, and they may feel guilty if they do not follow through on this [33]. However, this feeling is not always translated into purchase behavior. The literature implies that brilliant images sell better than “the sense of guilt” [33,34]. As value is never appreciated via guilt, it lacks meaning and purpose. Marketers should deliver positive images such as a “happy plant” when they are promoting green businesses to inform the customers that organic products are not only healthier but have additional benefits and are worth the premium [35]. Moreover, emotionally stirring information such as “simple things you can do to save the earth” or “small step, big changes” need to be communicated to consumers to encourage them (S-5). Current research indicates that although most participants seemed concerned about the environment, their concern did not translate into organic consumption. Those who purchase organic food are mainly driven by issues such as price, availability, and labeling of organic products. This indicates that marketers should focus on the communication of organic benefits to consumers to gain consumer trust [36]. Accordingly, creative communication strategies should be developed to overcome the barriers to consumption. The packaging design that promotes the organizational image plays a significant role in value distribution [37,38]. According to our interview-based research, consumers are not aware of professional information from producers. Their information is mostly from mass media. Thus, if there is a negative report in the media, it will affect their confidence in consuming organic products.

#### 4.2.2. Education

**Figure 11:**
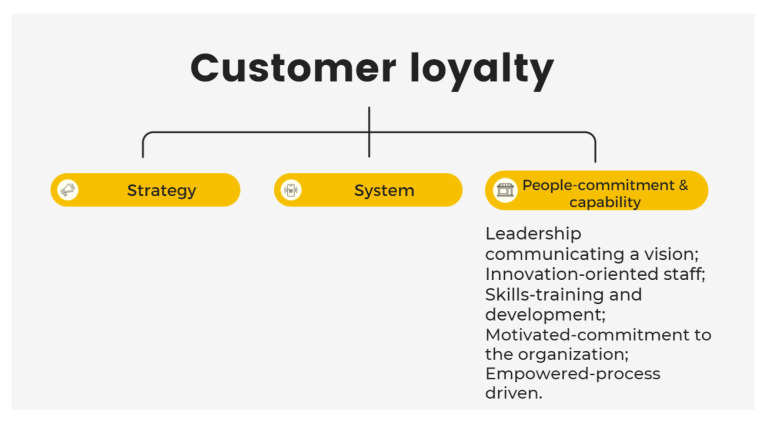
Market-led innovation: people-commitment and capability [18].

The “people” element is proposed to discuss how a vision can be communicated through leaders and staff to customers and how much customers understand the products with the theme of education (shown in Figure 11). The results of the interviews indicate that respondents have little understanding of organic products and believe that organic products are in general natural, healthier, and good for the environment, and follow strict rules that make them different from other healthy or more natural products [39]. However, if consumers do not realize their true value, they will question why they need to purchase them. As C-5 argued, arguments for organic food may affect her confidence in purchasing it. C-8 considers that the issue of E. Coli may stop him from buying, and a stronger reason is required to persuade him to pay a premium for organic food. These findings indicate the necessity of educating consumers. The literature supports the argument that consumers will pay a premium if they know a product is cost-effective in the long term [40]. However, knowing the benefits of organic food and educating the target segment is critical. As C-1 believes: “Only organic consumers know the value.” Marketers should work on promoting comprehensive knowledge of organic products through education such that the bright side of organic products such as assurance of safety, happiness, and enjoyment can be appreciated. 

Doyle and Bridgewater’s market-led innovation theory that promotes the use of the commitment and capabilities of organizational staff can be employed as a solution here [18]. In this theory, the development of the organization’s systems and implementation of its strategy relies on the commitment and skills of its staff; it is the key element of organizational effectiveness. The staff need to be trained to deliver the company’s vision to customers. As S-2 noted, their goal is to take care of people’s health by talking to people and marketing locally. Undoubtedly, the duties of an organic business include educating consumers about the vision of the organic future [41,42]. Both suppliers and consumers agree with the necessity of education that comprises not only imparting professional knowledge, but also concepts and ideas. According to our findings, some consumers doubt whether the cost of organic products is worth the premium and the value promised. Some advertisements overpromote organic products. It may attract consumers in the beginning, but consumers may end up losing confidence if they do not get what they expect. Honesty is the best policy in this case [43,44].

#### 4.2.3. Dynamic Customer Focus

**Figure 12:**
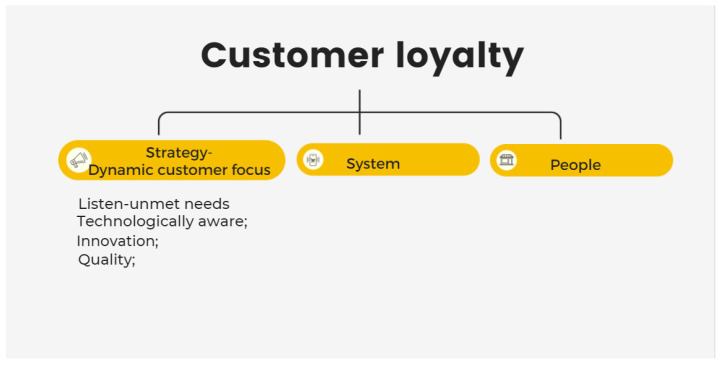
Market-led innovation: strategy [18].

The element of strategy in Figure 12 is discussed alongside the theme of dynamic customer focus to communicate how to fulfill unmet needs, technologically aware, innovation, and quality. Although positive and negative opinions regarding organic food remain diverse, specifically targeting market segments may provide opportunities for success. Trying to satisfy everyone is a marketing disaster [45]. According to C-1 and C-12, both organic food consumers, organic-product customers are healthier and more affluent, educated, and eco-friendly. They believe that health and quality are more important than taste and price. C-1 and C-12 are in teaching; C-12 purchases organic products for their health benefits, while C-1 aims to become healthier by consuming organic food. Therefore, customers who share these similarities may be a potential segment for organic products. By matching brand/product images, the correct approaches to attract the right consumers more effectively can be identified [46].

### 4.3. Summary

**Figure 13:**
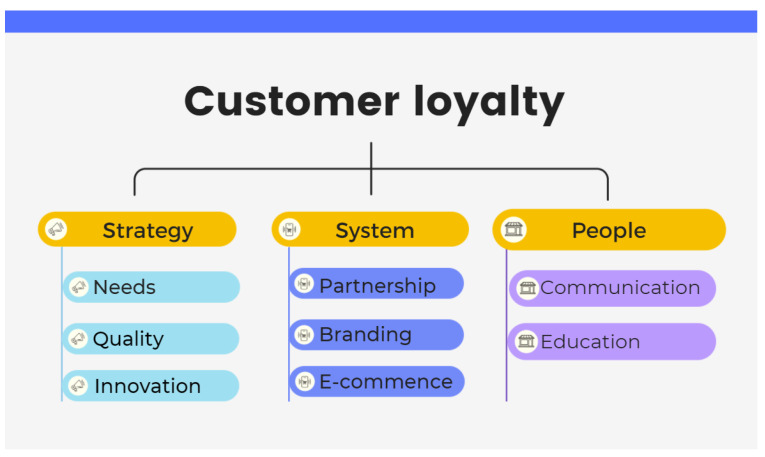
The framework of market-led innovation for organic food (organized by the authors based on the framework of Doyle and Bridgewater, 2012 [18]).

The secret of profitability is to retain customer loyalty while attracting new customers. The organic food business is no exception to this. This study used a market-led innovation framework and applied it to the organic food business. The content of three pillars—strategy, system, and people—were enriched by the results of this study shown as Figure 13. First, marketers should offer quality products or services to win customers’ satisfaction and retain customer loyalty. This framework suggests the element of strategy to fulfill dynamic customer focus, through identifying unmet needs with quality products and constant innovations, as organic food customers usually expect quality products with a variety of choice and taste. Second, the system of value delivery can use not only the power of partnership and branding stores, but also the disruptive capability of new technologies to offer the value of availability. Based on this, new technologies such as e-commerce and social media promotion can help with an effective organization. Third, how to communicate a products’ vision and educate customers should be the focus of people’s commitment and capability in the organic food business. 

### 4.4. Implications and Recommendations 

Developing partnerships and branding may help increase the publicity of products. E-commerce and logistic system formulation and improvement are recommended to remove obstacles to availability. Increasing the appeal and educating customers on the benefits of consuming organic food may help overcome the barrier of price; once they see the value of the products, they may be willing to pay a premium. The results suggest that the segment of customers, who have more affluence, higher education, better health, and eco-awareness, are more willing and can afford to purchase organic products. Marketers should focus on appropriate segmentation for effective marketing. Producers must engage in a more proactive approach in communicating with customers. Honestly informing consumers of the advantages and disadvantages of organic products may be the best way in the long run. Some potential consumers may be interested in organic products; however, the premium price may prevent them from purchasing, or they may believe that only the upper-medium class will go organic. Marketers need to encourage potential customers to keep an open mind for organic products. Affordability is always a matter of purchase behavior. Nevertheless, some consumers may not buy all organic materials, but they may start their organic experience via small purchases. Thus, the marketer should educate customers that everyone can “go organic”. This suggests that eating habits and preferences are prone to change. Marketers need to carefully observe consumers, as many opportunities may emerge at any time. Therefore, more creative inputs are required to innovate marketing strategies for business success.

### 4.5. Limitations and Future Study

The research could not cover the entire region of Northern Ireland because of time and travel constraints. The non-inclusion of major retail stores, due to their inability to put forward a spokesperson, could be viewed as a limitation; however, the multinational stores usually have in place a well-thought-out and well-researched marketing strategy. Although various perspectives were explored to enrich this study, this study is limited to those who were willing to agree to be interviewed. Despite the limited interview samples, the results offer a preliminary foundation for formulating a large-scale survey. The factors, motivations, and barriers identified can build up the content of quantitative-based questionnaires. Combined with qualitative study approaches, a mixed-method study can be developed accordingly to enrich and even enhance the content of organic food marketing. The framework for the organic food business can be further developed and modified as a theoretic model based on the outcomes of quantitative studies or mixed-method studies in future research.

## 5. Conclusions

This pilot study identified factors, motivations, and barriers to purchasing organic food to explore how market-led innovation strategy can fuel organic products business growth. The factors that persuade customers to purchase organic products are convenience, availability, variety, quality, brand and source, price, health, taste, and environmental friendliness, while the barriers to their purchase are premium price, availability, and lack of appeal. Results suggest that consumers do not need to be protected from the reality of organic food. The recommendation for marketers is to enhance the education of consumers and to pursue clear communication with them, as honesty may be the best policy here. Customers’ loyalty lies in trust, not in the brand. The results also show that availability, variety, and taste are the top three factors affecting consumers’ purchase decisions. Surprisingly, price or health consciousness is not the first concern for consumers; moreover, and contrary to general beliefs, the concept of health and socio-economic or even ecological awareness determine customers’ purchasing behavior. To conclude, customers appreciate the value delivered from the idea of “organic” rather than the product itself. Overall, the positive image of organic food contributes more to sales. Moreover, the study suggests that market-led innovation can be a good mechanism to enable organic products business growth through its three pillars of strategy, system, and people to achieve customer loyalty. Marketers are encouraged to deliver their value propositions based on the inner needs of their target customers for sharing the organic future together with them. 

## Figures and Tables

**Table 1:** List of interviewees * in the meetings.

Meeting	Type	Age (years)	Sex	Industry and Position
			(M/F)	
1.	C-1, Organic food consumer/ Obesity problem	39	Male	PhD student at the University of Ulster/Lecturer
2.	S-1, Food supplier	45	Male	Sales manager for Northern Ireland, McColgan’s Quality Foods
3.	C-2, Non-organic food consumer	40	Female	Householder, nursing background
4.	S-2, Food supplier/Local farm shop	44	Male	Owner of Gentender Ltd.
5.	C-3, Health food consumer	24	Female	A social worker from Rathbone, Belfast
6.	C-4, Health food lover/ Consumer/ Vegetarian	25	Female	Charity administrator
7.	C-5, Potential organic food consumer	55	Female	Senior secondary school teacher
8.	C-6, C-7, Non-organic food consumer/ Obesity and chronic diseases	50 58	Female Male	Nurse Teacher (this was a couple)
9.	S-3, Food supplier	50	Male	Ireland commercial manager at Dale Farm Ltd., Belfast, UK
10.	C-8, Organic and health food consumer	26	Male	PhD student at the University of Ulster (European surveillance of congenital anomalies benefit risk analysis in food)
11.	C-9, Organic food consumer	39	Female	Landscape business director, self-employed
12.	E-1, E-2, Organic business expert	40/23	Male	Agriculture and rural development/ Food standard agency
13.	P-1, Organic food producer S-4, Organic food supplier	42/ 43	Male/ Male	Owner of Burren Organic Farm Marketer at Burren Organic Farm
14.	S-5, Organic food supplier	58	Female	Organic products store manager
15.	C-10, Potential consumer	36	Male	Fitness instructor
16.	C-11, Potential consumer	42	Male	PhD, University lecturer
17.	C-12, Organic consumer	58	Female	Vice-chancellor in a primary school

* S, supplier; C, customers; P, producer; E, expert.

**Table 2:** Consumers’ background information.

	Age	Sex	Family Status	Employment Status	Health Status	Organic Food Consumers	Nationality
C-1	39	M	Married w/1 child	PhD/ University Lecturer	Obesity	Yes	Nigeria
C-2	40	F	Married w/1 child	Household/ Nursing background	OK	No	UK
C-3	24	F	Single	Social worker	Good	Casually	Germany
C-4	25	F	Single with partner	Administrator	Good	Casually	UK, British born Turkish,
C-5	55	F	Married w/1 child	Senior secondary school teacher	Good	Yes	UK
C-6	50	F	Married w/2 child	Teaching Assistant/ Nursing background	Good	Casually	UK
C-7	58	M	Married w/2 child	Part-time teacher	Obesity	No	UK
C-8	26	M	Single	PhD student	Good	Yes	Dutch
C-9	39	F	Married no child	Self-employment	Good	Yes	UK
C-10	36	M	Single	Fitness instructor	Good	Rarely	UK
C-11	42	M	Single	PhD/University Lecturer	Good	Rarely	Iran
C-12	58	M	Married w/1 child	Retired businessman	Normal, Cancer recovered	Rarely	UK

**Table 3:** Frequencies of response themes.

Ranked	Q1. Influenced Factors	Q2. Motivation	Q3. Barriers	Q4. Eco-Concern	Q5. Knowledge
1.	Availability	Tasty	Availability	Yes	Insufficient
2.	Variety	Health concept	Culture: eating habits		
3.	Taste	Eco-friendly	Price		
4.	Quality	Cost-effective	Insufficient knowledge of organic food		
5.	Brand, Source	Health concept	Value of price		
6.	Price	Food security	Source		
7.	Health-conscious, environmentally friendly				

**Table 4:** Analysis of research questions for suppliers.

Ranked		Q1. Motivation	Q2. Barriers	Q3. Eco. Concern	Q4. Knowledge
1.	Availability		Strategy	Yes	Insufficient
2.	Variety		Price, culture, eating habits, knowledge of organic food	Yes	Insufficient
3.	Taste, quality		Taste, high cost, capability	Yes	Insufficient
4.	Brand, source		Price	Yes	Insufficient
5.	Health concept, profit		Knowledge and price	Yes	Insufficient

**Table 5:** Factors affecting consumers purchasing decisions.

**Questions for** **Research**	**Why Do Customers Buy the Goods or Services You Provide?**	**What Do Customers Value?**	**How Could That** **Value Be Delivered?**	**Literature Applied**
Finding	-Convenience/ Availability -Variety -Taste -Quality -Brand/Source -Price -Health -Eco-issue/ Social responsibility	-Efficiency/Consideration -Pleasure -Trust/Assurance/Relief -Value -Sustainable happiness -Long-term sustainability	-Availability (Place) -Communication (Promotion) -Education (Product)	Market-led innovation (Doyle Bridgewater, 2012); The innovator’s solution (Christensen, 2003).
Discussion	Availability	Branding	Communication	

## Data Availability

Not applicable.
